# Shapley additive explanations based feature selection reveals CXCL14 as a key immune-related gene in predicting idiopathic pulmonary fibrosis

**DOI:** 10.3389/fmed.2025.1608078

**Published:** 2025-08-06

**Authors:** Bin Chen, Lu Huan, Junyu Lu, Jinhe Yuan

**Affiliations:** ^1^Department of Geriatric Palliative Medicine, Chongqing Liangjiang New District People Hospital, Chongqing, China; ^2^Department of Respiratory and Critical Care Medicine, Renji Hospital, School of Medicine, Chongqing University, Chongqing, China

**Keywords:** idiopathic pulmonary fibrosis, gene expression, machine learning, immune cell infiltration, Shapley additive explanations

## Abstract

**Background:**

Idiopathic pulmonary fibrosis (IPF) is a progressive lung disease marked by excessive fibrous tissue accumulation in the lung interstitium, leading to a gradual deterioration of respiratory function and significantly impairing patients’ quality of life. Despite advances in understanding its etiology and pathogenesis, the exact mechanisms remain unclear, underscoring the need for novel biomarkers and therapeutic targets.

**Methods:**

We analyzed five publicly available datasets from the Gene Expression Omnibus (GEO), specifically “GSE15197,” “GSE53845,” “GSE135065,” “GSE185691,” and “GSE195770,” to identify gene expression changes associated with IPF. Data were annotated and normalized to minimize batch effects and technical variability. Principal Component Analysis (PCA) verified preprocessing efficacy. Differentially expressed genes (DEGs) were identified using linear modeling. Core DEGs were selected via integrative analysis across datasets.

**Results:**

Our analysis revealed DEGs that are substantially linked to crucial biological processes such as extracellular matrix organization and immune response regulation. Integrative analysis of five GEO datasets identified CXCL14, MMP7, and MDK as core differentially expressed genes in the final predictive model. Using Least Absolute Shrinkage and Selection Operator (LASSO) regression and Random Forest, we constructed a logistic regression model with robust predictive performance, achieving an AUC of 0.92 in the training cohort and 0.89 in the validation cohort, with sensitivity of 88% and specificity of 85%. The Shapley Additive Explanations (SHAP) method identified CXCL14 (mean SHAP value = 0.38) as the most influential feature, followed by MMP7 and MDK. Functional enrichment analyses highlighted significant enrichment of TGF-*β* signaling, extracellular matrix organization, and chemokine signaling pathways. Immune infiltration analysis revealed positive correlations between CXCL14 expression and alveolar macrophage/activated fibroblast populations, while SHAP interaction analysis identified synergistic effects between CXCL14 and TGF-β1 in driving fibrosis.

**Conclusion:**

These findings substantiate the hypothesis that IPF pathogenesis is closely linked to extracellular matrix remodeling and immune dysregulation. This suggests that future investigations should delve deeper into the practical applications of identified biomarkers in the early diagnosis and management of IPF. Furthermore, the machine learning-based predictive model demonstrates strong clinical potential and merits further validation in prospective trials to assess its utility and therapeutic implications in real-world settings.

## 1 Introduction

Idiopathic Pulmonary Fibrosis (IPF) represents a progressive, fatal interstitial lung disorder characterized by aberrant pulmonary tissue fibrogenesis and irreversible decline in respiratory function, with a median survival duration of merely 3–5 years (1). Despite the clinical approval of tyrosine kinase inhibitors (e.g., Nintedanib) and antifibrotic agents (e.g., Pirfenidone), substantial interindividual variability in therapeutic responses persists: approximately 30% of patients exhibit rapid disease progression post-pharmacotherapy, while treatment discontinuation rates due to adverse events reach 15–30% (2). Such heterogeneity in treatment outcomes underscores the dynamically complex and incompletely characterized molecular mechanisms of IPF. Current investigations predominantly focus on single-biomarker approaches (e.g., MMP7, surfactant protein D), yet their predictive utility demonstrates marked inconsistency across independent cohorts, thereby impeding the development of personalized treatment algorithms (3). Of particular note, emerging evidence has implicated CXCL14 (C-X-C chemokine ligand 14) in both fibrogenic pathways and immune dysregulation in IPF; however, its mechanistic roles and predictive value in clinical contexts remain poorly understood. Consequently, the systematic identification of multidimensional molecular signatures capable of accurately predicting treatment responses and the elucidation of their underlying mechanisms constitute critical scientific imperatives for improving IPF prognosis.

In recent years, research on the heterogeneity of IPF has achieved some breakthroughs: genomic studies have identified gene mutations such as Telomerase mutations (TERT) (4) and Mucin 5B (MUC5B) (5) as being associated with disease risk, proteomic studies have found that CXCL13 and CCL18 are related to the rate of decline in lung function (6), and single-cell sequencing techniques have revealed the central role of abnormally activated fibroblast subgroups in the fibrosis process (7). However, there are three key deficiencies in the existing achievements: firstly, most studies remain at the level of describing correlations and lack the verification of the causal relationship between biomarkers and treatment response; secondly, traditional statistical methods are difficult to integrate the non-linear interactions between high-dimensional omics data (such as transcriptomics, methylomics, and immunogenomics) and clinical parameters, leading to insufficient generalization capability of predictive models (8); what is more prominent is that although existing machine learning models can improve prediction accuracy, their “black box” nature hinders the interpretation of biological significance - for example, the Gradient Boosting Tree (XGBoost) model can predict the risk of treatment failure, but cannot answer which immune cell subtypes or signaling pathways drive resistance (9, 10). In addition, the reprogramming mechanism of immune cells in the IPF microenvironment has been long neglected: recent studies suggest that regulatory T cells (Treg) (11) and macrophage polarization (12) may affect drug response, but these findings have not yet been translated into operational predictive indicators. Notably, while CXCL14 upregulation has been documented in IPF-derived lung fibroblasts, its role in immune modulation and potential as a prognostic biomarker remain systematically uncharacterized. These bottlenecks collectively hinder the development of precision medicine strategies for IPF (13).

The present study employs an integrative approach combining multi-omics data analysis, machine learning-based feature selection, and SHAP (Shapley Additive Explanations) interpretability analysis to systematically identify key molecular features and evaluate their utility in IPF personalized medicine. Specifically, this investigation aims to address the following knowledge gaps: (1) validate CXCL14 as a pivotal immune-related biomarker through SHAP-driven feature importance analysis; (2) decipher the mechanistic associations between CXCL14 expression and immune cell infiltration (e.g., Treg and macrophage polarization); and (3) develop an interpretable machine learning framework for predicting IPF progression based on CXCL14 and associated molecular signatures. To achieve these objectives, five datasets from the Gene Expression Omnibus (GEO) were subjected to rigorous preprocessing, including data normalization, batch effect correction via ComBat, and principal component analysis (PCA) to ensure inter-dataset consistency. Differential gene expression analysis (DEA) was subsequently performed to identify IPF-associated transcripts, which were further refined using LASSO (Least Absolute Shrinkage and Selection Operator) regression and random forest (RF) models to derive a robust set of core feature genes. SHAP analysis was employed to quantify the contribution of individual gene features to model predictions, thereby mitigating the interpretational limitations of traditional machine learning. Complementary immune infiltration analysis (CIBERSORT) and pathway enrichment analyses (GSEA, GSVA, KEGG) were conducted to characterize the functional roles of identified molecular features within the IPF microenvironment. By integrating mechanistic and predictive analyses, this study not only establishes a high-precision, interpretable model for IPF treatment response prediction but also positions CXCL14 as a novel therapeutic target by delineating its dual roles in fibrogenesis and immune dysregulation.

## 2 Methods

### 2.1 Dataset acquisition and preprocessing

This study utilized five publicly available datasets from the Gene Expression Omnibus database (GEO),^1^ including GSE15197, GSE53845, GSE135065, GSE185691, and GSE195770. These datasets encompassed transcriptomic profiles from lung tissue and immune cells derived from patients with IPF and normal controls. For improved clarity, key characteristics of each dataset were summarized in Table 1. “GSE15197” (8 IPF and 13 normal lung tissues) (14): Sample source: Lung tissues from patients at the Mayo Clinic and normal donors; Tissue type: Formalin-fixed paraffin-embedded (FFPE) lung biopsies; Disease stage: Mixed stages (early to advanced IPF); Sequencing platform: Affymetrix Human Genome U133 Plus 2.0 Array. “GSE53845” (40 IPF and 8 normal lung tissues) (15): Sample source: Lung tissues from the University of Michigan IPF cohort; Tissue type: Fresh-frozen lung parenchyma; Disease stage: Advanced IPF (confirmed by high-resolution computed tomography); Sequencing platform: Illumina HumanHT-12 v4 Expression BeadChip. “GSE135065” (9 IPF and 9 normal lung tissues) (16) Sample source: Bronchoalveolar lavage (BAL) fluid cells from IPF patients and healthy controls; Tissue type: Immune cells isolated from BAL fluid; Disease stage: Early-stage IPF (predominantly non-honeycombing fibrosis); Sequencing platform: RNA-seq (Illumina HiSeq 2,500, paired-end 100 bp). “GSE185691” (6 IPF and 8 normal lung tissues) (17) Sample source: Lung tissues from the Idiopathic Pulmonary Fibrosis Clinical Research Network (IPF-CRN); Tissue type: Laser-capture microdissected alveolar epithelial cells; Disease stage: Moderate IPF with mixed fibrosis and inflammation; Sequencing platform: Affymetrix Clariom S Human Array. “GSE195770” (4 IPF and 4 normal lung tissues) (18) Sample source: Lung fibroblasts derived from patient-derived explant cultures; Tissue type: Primary lung fibroblast cells; Disease stage: End-stage IPF (post-lung transplantation samples); Sequencing platform: RNA-seq (Illumina NovaSeq 6,000, 150 bp paired-end). These datasets include gene expression data relevant to IPF. The data processing steps are as follows: Data Annotation: The raw data were annotated using appropriate annotation files to ensure consistency between gene identifiers and gene names. This was performed using the biomaRt package. Data Normalization: Gene expression data were normalized using the normalizeBetweenArrays function from the limma package to eliminate batch effects and technical biases. Batch Effect Correction: To further address batch effects, we applied the ComBat method from the SVA package for batch effect correction, using “GSE135065” (16) as the validation dataset. PCA: PCA was conducted on the normalized data to assess the overall structure and verify the removal of batch effects. PCA plots were used to visualize the differences between datasets.

**Table 1:** Summary of GEO datasets used in this study.

Dataset	IPF/normal	Tissue type	Disease stage	Sample source	Platform
GSE15197	8 / 13	FFPE lung biopsies	Mixed	Mayo Clinic and normal donors	Affymetrix U133 Plus 2.0
GSE53845	40 / 8	Fresh-frozen lung parenchyma	Advanced	University of Michigan IPF cohort	Illumina HumanHT-12 v4
GSE135065 (validation dataset)	9 / 9	BAL fluid immune cells	Early	BAL fluid from patients & healthy controls	RNA-seq (Illumina HiSeq 2,500)
GSE185691	6 / 8	Microdissected alveolar epithelial cells	Moderate	IPF Clinical Research Network	Affymetrix Clariom S
GSE195770	4 / 4	Lung fibroblasts (explant cultures)	End-stage	Post-lung transplantation samples	RNA-seq (Illumina NovaSeq 6,000)

IPF, Idiopathic pulmonary fibrosis; FFPE, Formalin-fixed paraffin-embedded.

### 2.2 Differential gene expression analysis

Differential gene expression was performed using the limma package (19), which calculates gene expression differences between different groups (IPF group vs. normal control group) through linear modeling. Genes were considered significantly differentially expressed with a false discovery rate (FDR) < 0.05 and |logFC| > 1.

### 2.3 Functional enrichment analysis

Gene Ontology (GO) Enrichment (20): GO enrichment analysis was performed on the differentially expressed genes using the ClusterProfiler package (21), focusing on biological processes, molecular functions, and cellular components.

Kyoto Encyclopedia of Genes and Genomes (KEGG) Pathway Analysis (20): KEGG pathway enrichment analysis was also conducted using the ClusterProfiler package (22)to identify key pathways associated with IPF.

### 2.4 Nomogram construction

LASSO Regression (23): LASSO regression was applied to the differentially expressed genes to select features that most significantly predict IPF. Cross-validation was used to determine the optimal penalty parameter, thereby reducing overfitting.

Logistic Regression (24): The genes selected by LASSO regression were used as independent variables to construct a logistic regression model for predicting IPF occurrence. The output of the model provided the probability of each sample belonging to the IPF group. A nomogram for IPF prediction was constructed using the rms package (25), integrating the results from LASSO and logistic regression. This visual tool highlights the relative contribution of each differential gene to IPF prediction, providing an individualized prediction model.

### 2.5 Machine learning model construction and feature gene selection

To identify key feature genes associated with IPF and build a predictive model, we prioritized the use of both LASSO and RF (23) methods, which have complementary characteristics. LASSO emphasizes feature sparsity and global linear feature selection, while RF focuses on the importance of variables and the ability to recognize non-linear relationships. The joint use of both methods enables a more comprehensive identification of highly predictive genes, thus enhancing the model’s generalization and accuracy.

LASSO Regression: Using the glmnet package (26), LASSO regression was performed on the differential gene set, with 10-fold cross-validation to determine the optimal *λ* value, identifying the most significant genes associated with IPF. This method helps reduce model complexity and overfitting, highlighting the most diagnostically relevant genes.

RF: The random Forest package was used to rank feature importance within the differential gene set. Each gene’s “Mean Decrease Gini” value was computed to identify the genes most contributing to IPF prediction. RF excels in identifying non-linear relationships and uncovering complex gene interactions.

Intersection of Feature Genes: The genes selected by both LASSO regression and RF were intersected to derive a more robust core set of feature genes. These genes were used for subsequent modeling and biological analysis.

### 2.6 SHAP explainability analysis

To interpret the machine learning models, we used the SHAP method, implemented through the shap package, to provide explainability for the trained Support Vector Machine (SVM) and Random Forest models. SHAP values quantify the contribution of each feature gene to the model’s prediction, providing interpretability by attributing importance scores that indicate how much each gene influences the probability of IPF classification. This overcomes the ‘black box’ limitation of traditional machine learning by revealing the direction and magnitude of each feature’s impact. SHAP values indicate the importance and contribution of each feature gene in predicting IPF.

### 2.7 Enrichment and immune cell infiltration analysis

Gene Set Enrichment Analysis (GSEA): GSEA was performed on the differentially expressed genes using the Hallmark gene sets to explore key biological pathways associated with IPF (27).

Gene Set Variation Analysis (28) (GSVA): GSVA was used to score gene sets for each sample, allowing for a more detailed analysis of differences between IPF and normal samples.

Immune cell infiltration was estimated using the CIBERSORT tool (29) with the LM22 signature matrix, which comprises 547 gene signatures representing 22 immune cell subsets within the human white blood cell population, as derived from HGU133A microarray analysis. The analysis was conducted with 1,000 permutations and quantile normalization (QN = TRUE). This was used to analyze the RNA-seq data of different subgroups of IPF patients, to infer the relative proportions of 22 immune infiltrating cells, and to perform Pearson correlation analysis on gene expression and immune cell content. *p* < 0.05 was considered statistically significant. Furthermore, based on the largest pharmacogenomics database, named as Genomics of Drug Sensitivity in Cancer (GDSC),^2^ we used the pRRophetic package (30) to predict the chemosensitivity of each tumor sample.

## 3 Results

### 3.1 Differential gene expression and batch effect correction

**Figure 1:**
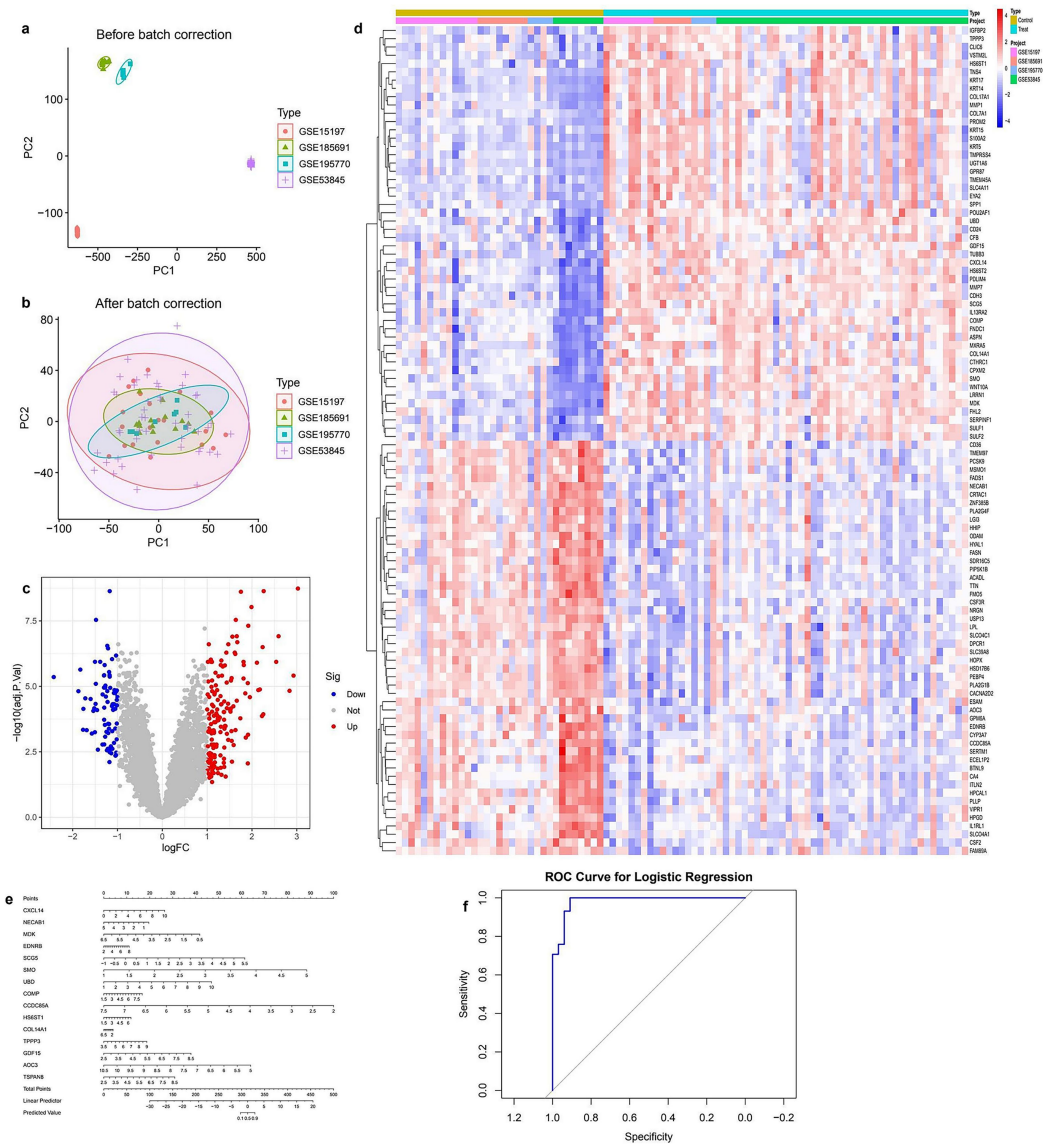
Integrated analysis of gene expression data from multiple GEO datasets **(a)** PCA plot before batch correction showing clear separation between datasets. **(b)** PCA plot after batch correction indicating effective removal of batch effects. **(c)** Volcano plot of differentially expressed genes; red and blue dots represent significantly upregulated and downregulated genes, respectively. **(d)** Heatmap of top differentially expressed genes across all samples, grouped by condition and dataset. **(e)** Nomogram based on key gene signatures for predicting disease risk. **(f)** ROC curve of the logistic regression model demonstrating strong classification performance.

To ensure data consistency across multiple datasets, batch effect correction was performed. PCA before correction revealed distinct clustering among different datasets, indicating substantial batch effects (Figure 1a). After batch effect removal, PCA analysis demonstrated a more homogeneous distribution of samples, suggesting effective normalization (Figure 1b). Differential gene expression analysis identified 1,237 significantly dysregulated genes (FDR < 0.05, |logFC| > 1), including 789 upregulated and 448 downregulated genes (Figure 1c), with full details provided in Supplementary Table S1. The heatmap of the top differentially expressed genes provided an overview of expression patterns across different sample groups, with red and blue indicating upregulated and downregulated genes, respectively (Figure 1d). To further investigate the predictive potential of these differentially expressed genes (DEGs), a logistic regression model was developed. The nomogram visualization illustrated the contribution of individual genes to the predictive model (Figure 1e). The model’s performance was assessed using Receiver Operating Characteristic (ROC) analysis, which demonstrated a high area under the curve (AUC), indicating strong discriminatory power (Figure 1f).

### 3.2 GO functional enrichment analysis of differentially expressed genes

**Figure 2:**
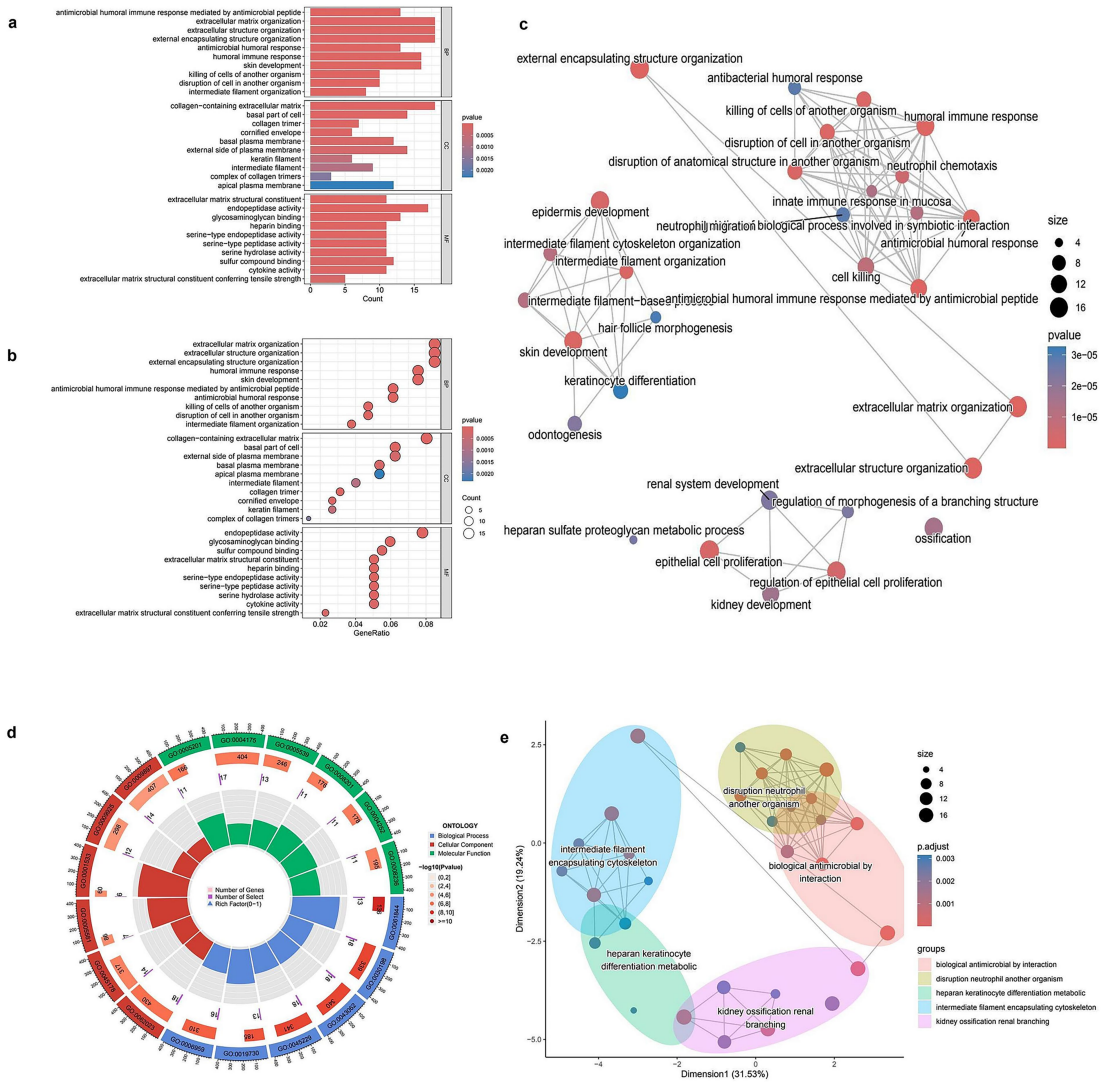
GO enrichment analysis of differentially expressed genes (DEGs). **(a)** Bar plot showing the top enriched Gene Ontology (GO) biological processes among DEGs. Bar length represents gene count, and color indicates statistical significance (*p*-value). **(b)** Bubble plot of enriched GO terms. The x-axis shows gene ratio, bubble size indicates gene count, and color reflects p-value. **(c)** GO term network showing relationships between enriched biological processes. Node size corresponds to the number of genes involved; color indicates adjusted p-value. **(d)** Circular visualization of GO terms categorized by function. Inner rings display the number of genes and significance of each term. **(e)** Multidimensional scaling (MDS) plot grouping enriched GO terms into clusters based on semantic similarity. Each color-coded cluster represents functionally related biological processes.

To investigate the biological significance of DEGs, GO enrichment analysis was conducted. The bar plot of biological processes (BP) enrichment highlighted key pathways associated with DEGs, including extracellular matrix organization, antimicrobial humoral response, and collagen metabolic processes, with significant terms ranked by gene count and *p*-value (Figure 2a). Similarly, the dot plot representation provided an alternative visualization of GO enrichment, where the size of each dot corresponded to the number of genes involved in a specific function, and color intensity indicated statistical significance (Figure 2b). The GO network plot illustrated the functional relationships among enriched biological processes, showing clusters of interconnected pathways related to immune response, extracellular structure organization, and epithelial development (Figure 2c). To further categorize the identified GO terms, a circular plot visualization was generated, displaying the distribution of DEGs across three major GO domains: biological processes (BP), molecular functions (MF), and cellular components (CC; Figure 2d). This classification provided a comprehensive overview of DEG involvement in different cellular activities. Lastly, a dimensional reduction clustering plot grouped functionally related GO terms into distinct categories, revealing major clusters associated with extracellular matrix remodeling, immune-related defense mechanisms, and epithelial differentiation (Figure 2e). These results indicate the major biological pathways in which DEGs are involved, highlighting their roles in structural organization and immune modulation (Full details provided in Supplementary Table S2).

### 3.3 KEGG functional enrichment analysis of differentially expressed genes

**Figure 3:**
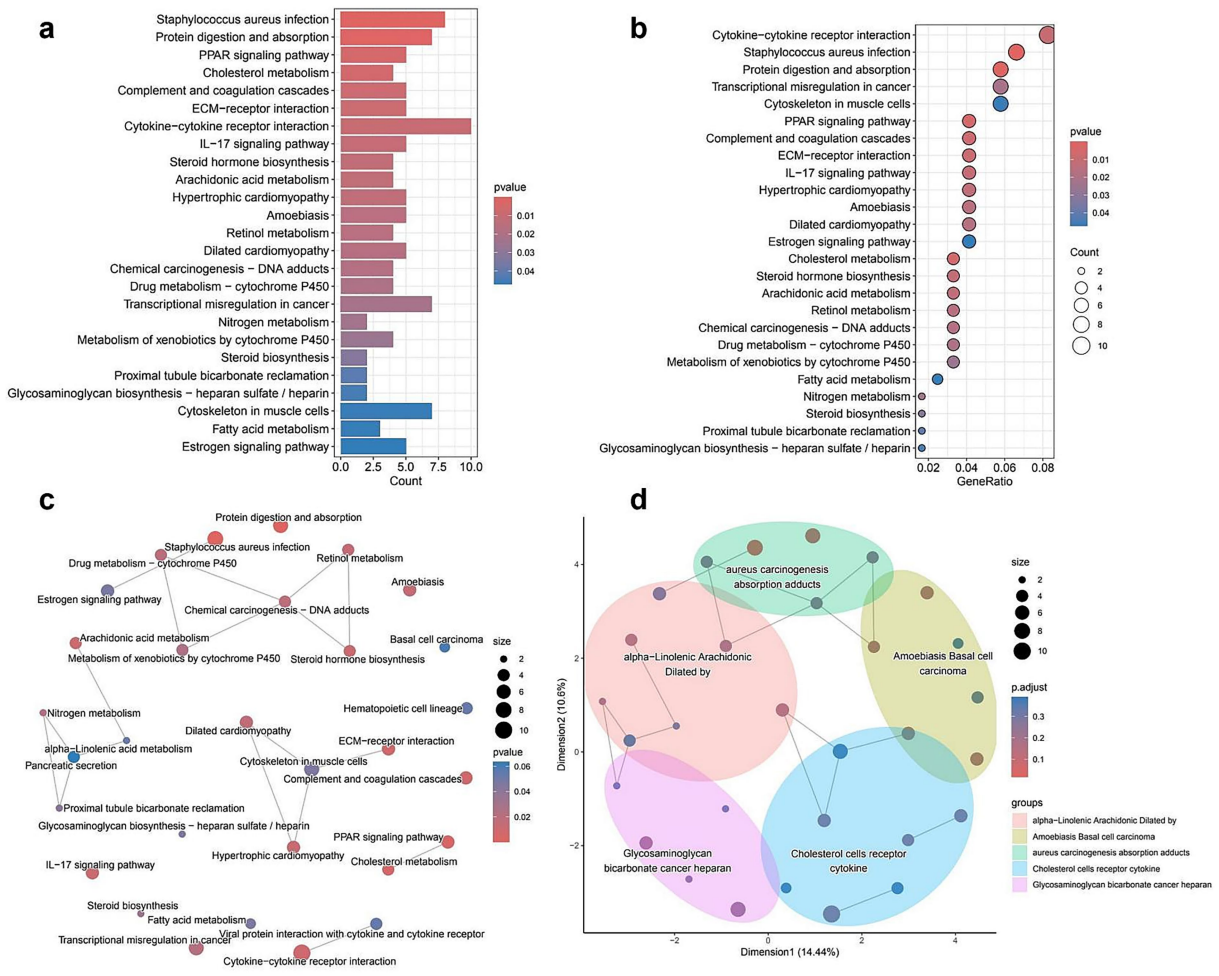
KEGG pathway enrichment analysis of differentially expressed genes (DEGs). **(a)** Bar plot of the top enriched KEGG pathways. Bar length represents the number of DEGs involved in each pathway, and color shading reflects statistical significance (*p*-value). **(b)** Bubble plot showing the relationship between gene ratio and enrichment significance. Larger bubbles indicate more genes involved in a given pathway; color gradient shows *p*-value. **(c)** Network diagram of enriched pathways. Nodes represent KEGG pathways; node size indicates gene count, and color shows *p*-value. Edges represent functional similarities between pathways. **(d)** Semantic similarity map clustering related pathways into functionally grouped modules. Each colored region represents a cluster of biologically similar pathways; dot size represents the number of genes, and color reflects adjusted *p*-values.

The KEGG pathway enrichment analysis of DEGs revealed significant associations with various biological processes and disease-related pathways. The bar plot (Figure 3a) illustrates the most enriched pathways, with *Staphylococcus aureus* infection, protein digestion and absorption, and Peroxisome Proliferator-Activated Receptor signaling pathway ranking among the top. The color gradient represents statistical significance, with lower *p*-values indicating stronger enrichment. In the dot plot (Figure 3b), the gene ratio is plotted against pathway categories, showing similar trends, where cytokine–cytokine receptor interaction and complement and coagulation cascades display high enrichment scores. The size of the dots represents the number of genes involved, reinforcing the prominence of these pathways in the dataset. The network plot (Figure 3c) further explores the interconnectivity of enriched pathways, highlighting functional clusters such as immune response pathways (e.g., cytokine–cytokine receptor interaction) and metabolic processes (e.g., fatty acid metabolism, cholesterol metabolism; full details provided in Supplementary Table S3). Pathways with shared gene components are linked, providing insight into their potential regulatory interplay. Lastly, the enrichment map (Figure 3d) organizes pathways into broader functional clusters, visually grouping related biological processes. Key clusters include lipid metabolism (alpha-linolenic acid and arachidonic acid metabolism), immune system pathways (*Staphylococcus aureus* infection and cytokine signaling), and extracellular matrix interactions (Extracellular Matrix-receptor interaction and glycosaminoglycan biosynthesis). The distribution of pathways within distinct clusters underscores their functional relevance and highlights potential mechanistic relationships among different biological processes.

### 3.4 Gene selection and differential expression analysis

**Figure 4:**
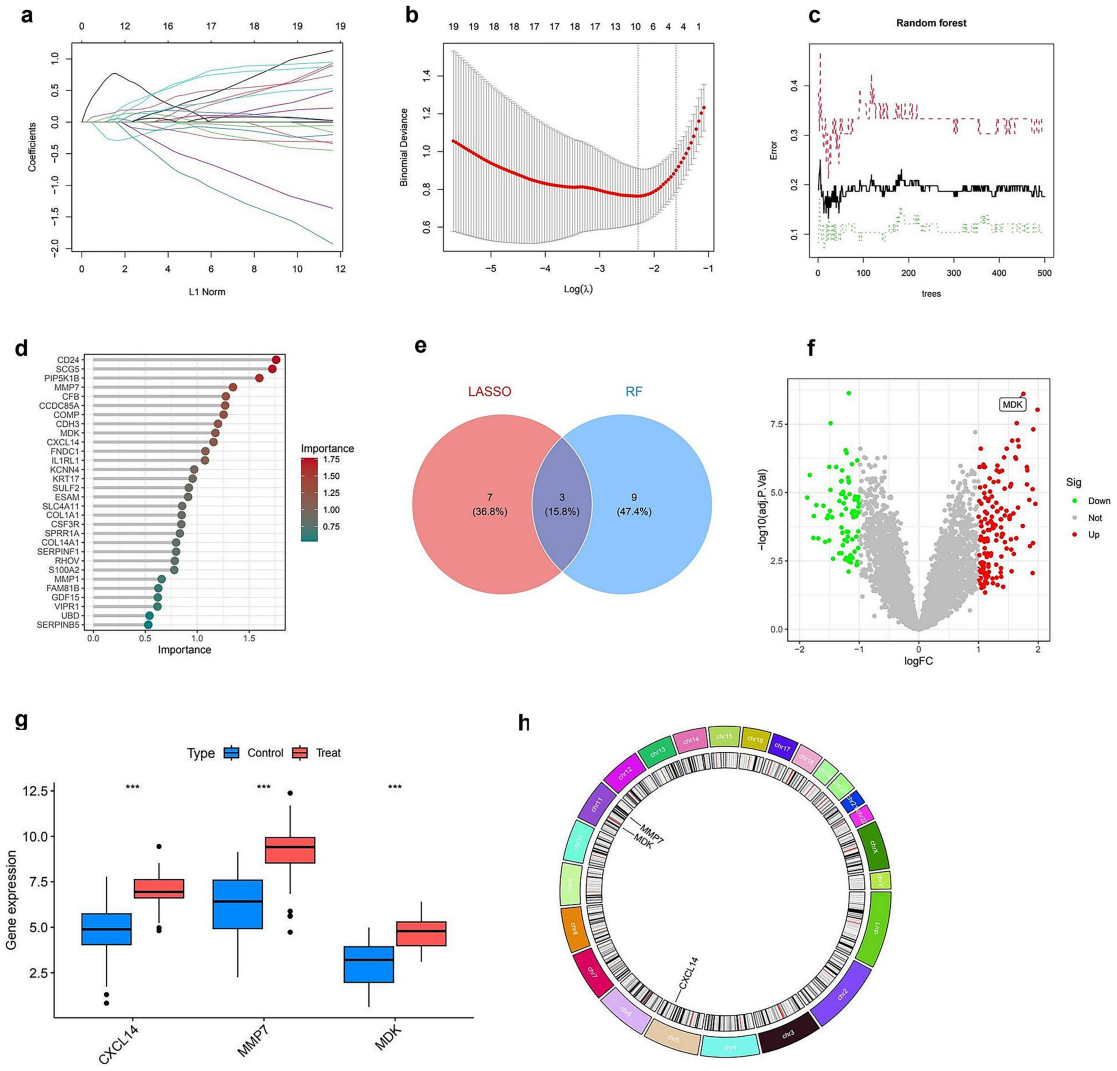
Identification and characterization of key diagnostic genes. **(a)** LASSO coefficient profiles of 19 genes plotted against the L1 norm. **(b)** Ten-fold cross-validation plot for optimal lambda selection in the LASSO model. The vertical dotted line indicates the value with minimum cross-validation error. **(c)** Random forest (RF) model error rates plotted against the number of decision trees. Black line shows overall error; green and red lines represent class-specific errors. **(d)** Variable importance ranking from the RF model; top genes contributing most to classification accuracy are shown with color gradient by importance score. **(e)** Venn diagram showing overlap of feature genes identified by LASSO and RF models; three genes (CXCL14, MMP7, and MDK) were shared by both methods. **(f)** Volcano plot showing differentially expressed genes. The three selected diagnostic genes are highlighted; MDK is labeled for emphasis. **(g)** Boxplots showing expression levels of CXCL14, MMP7, and MDK between control and treatment groups; all three genes are significantly upregulated in the treatment group (***p < 0.001). **(h)** Chromosomal locations of the three key diagnostic genes, visualized in a circos plot.

Figure 4 illustrates the integration of LASSO and random forest models to identify key genes and assess their differential expression. Panel (a) shows the effect of LASSO regularization on gene selection, with coefficients plotted against the L1 norm, indicating the genes retained at various regularization levels. Panel (b) demonstrates the cross-validation procedure used to determine the optimal regularization parameter (lambda) that minimizes error. The performance of the random forest model is illustrated in panel (c), where error rates are plotted as a function of the number of trees, stabilizing after a certain threshold. In panel (d), the importance of each gene is ranked according to the random forest model, highlighting the most influential variables. The Venn diagram in panel (e) compares the gene sets selected by LASSO and random forest, showing a partial overlap (seven common genes), with three genes uniquely selected by LASSO and nine by random forest. Panel (f) depicts a volcano plot of differentially expressed genes, with upregulated genes highlighted in red and downregulated genes in green, indicating significant changes in expression. Panel (g) presents boxplots of selected genes (CXCL14, MMP7, MDK) showing significant expression differences between control and treated groups. Finally, panel (h) presents a Circos plot visualizing the chromosomal locations of the selected genes, providing insight into their genomic distribution (Full details provided in Supplementary Table S4). These analyses collectively identify key genes that may play a role in the response to treatment.

### 3.5 SHAP-based feature importance and model performance evaluation

**Figure 5:**
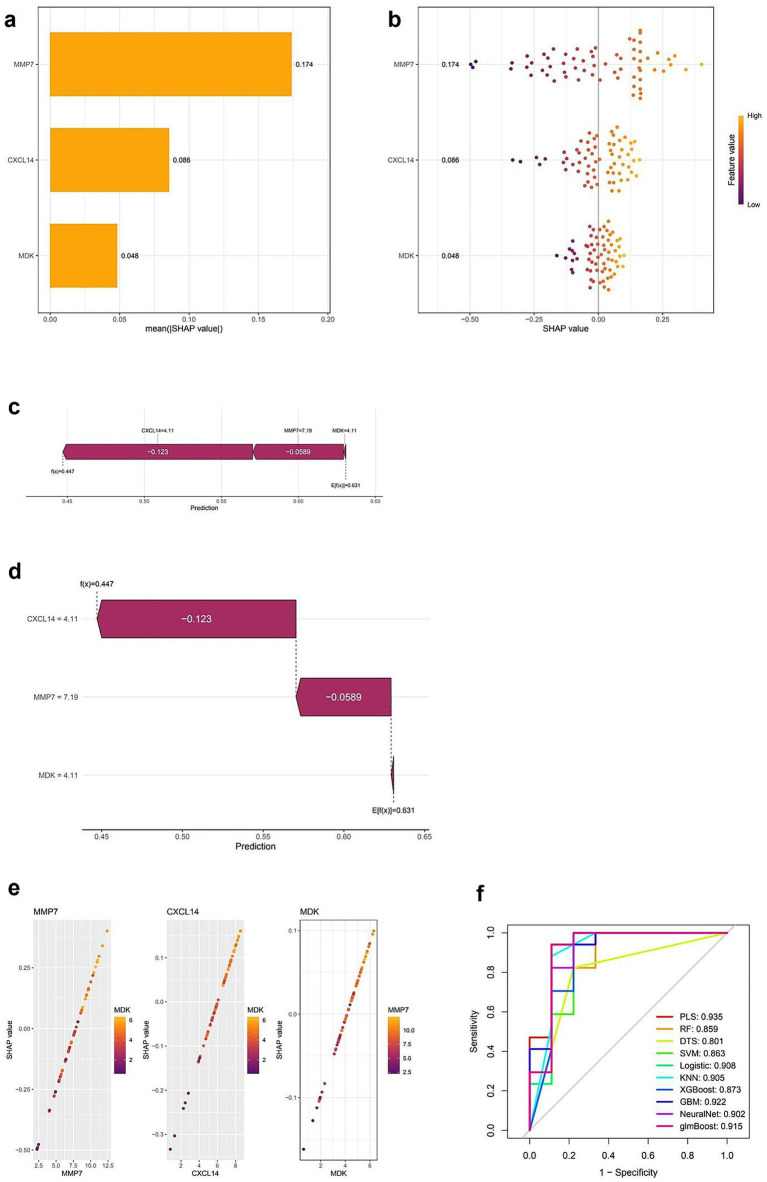
SHAP-based interpretation and model performance evaluation of key diagnostic genes. **(a)** Bar plot of average SHAP values showing the importance of MMP7, CXCL14, and MDK in the predictive model. MMP7 contributes the most to model output. **(b)** SHAP summary plot displaying the impact of each feature value on model output. Each dot represents a sample; color indicates feature value (purple = low, yellow = high). **(c,d)** SHAP force plots showing the individual prediction contributions of CXCL14, MMP7, and MDK. Red segments push the prediction higher, and blue segments push it lower. **(e)** SHAP dependence plots illustrating the relationship between SHAP values and expression levels for each gene. The interaction between genes (e.g., MMP7 and MDK) is also visualized. **(f)** ROC curves comparing different machine learning models for diagnostic classification. PLS (Partial Least Squares) achieves the highest AUC (0.935), followed by NeuralNet (0.902) and Logistic Regression (0.908), indicating strong model performance across various classifiers.

Figure 5 presents the SHAP-based interpretation of feature importance alongside model performance evaluation. Panel (a) displays a bar plot of mean SHAP values, indicating that MMP7 has the highest impact on model predictions, followed by CXCL14 and MDK. Panel (b) provides a SHAP summary plot, illustrating the distribution of SHAP values across individual predictions, with color gradients representing the feature values. Higher values of MMP7 are associated with positive SHAP values, suggesting a strong influence on the model’s output. Panels (c) and (d) present SHAP force plots that visualize individual prediction contributions, showing how each feature positively or negatively affects specific classification outcomes. Panel (e) consists of scatter plots depicting the correlation between SHAP values and feature values for MMP7, CXCL14, and MDK, where color intensities indicate the strength of feature importance. Finally, panel (f) displays ROC curves comparing the performance of multiple classification models, with AUC values ranging from 0.859 (random forest) to 0.935 (partial least squares, PLS). Neural network and logistic regression models also exhibit high AUC values, indicating robust classification performance (Full details provided in Supplementary Table S5).

### 3.6 Pathway enrichment and immune cell profiling

**Figure 6:**
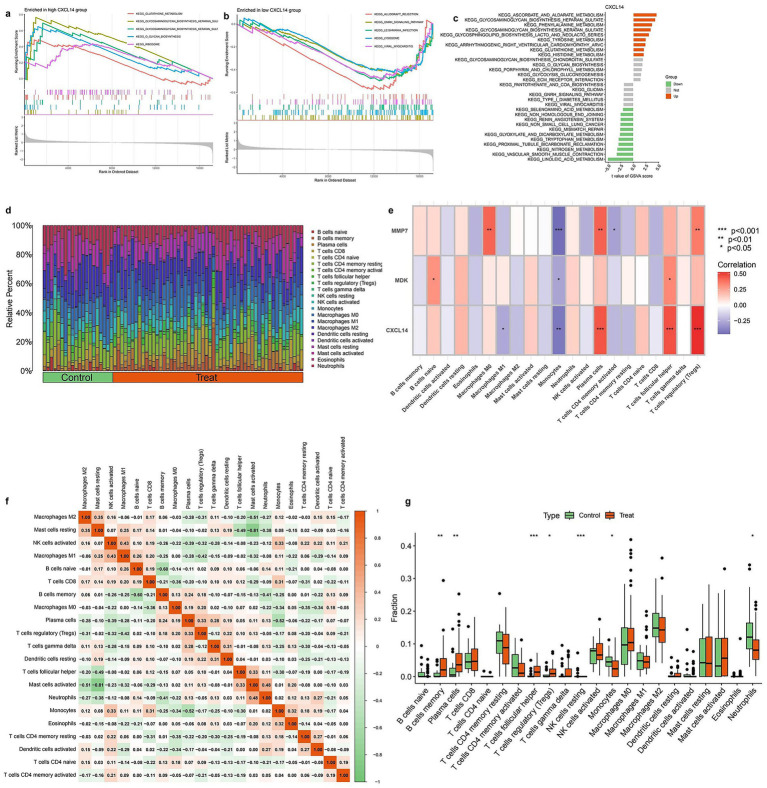
Immune infiltration analysis and correlation with diagnostic genes. **(a)** GSEA results showing KEGG pathway enrichment in the high CXCL14 expression group. Immune-related pathways such as “cytokine–cytokine receptor interaction” and “chemokine signaling pathway” are enriched. **(b)** GSEA plot of KEGG pathways enriched in the low CXCL14 group. Metabolic and signaling pathways appear more active in the low-expression group. **(c)** KEGG pathway enrichment bar plot for CXCL14 co-expressed genes. Immune-associated pathways are predominantly enriched in the high-expression group. **(d)** Stacked bar plot showing the relative proportions of 22 immune cell types in control and treated samples based on CIBERSORT analysis. Notable differences in T cells, macrophages, and dendritic cells are observed between groups. **(e)** Heatmap showing the correlation between expression of diagnostic genes (MMP7, MDK, CXCL14) and immune cell infiltration levels. Strong positive correlations are observed with M2 macrophages and Tregs. Significance is indicated (*p < 0.05, **p < 0.01, ***p < 0.001). **(f)** Correlation matrix among immune cell types. Strong negative correlations are seen between M2 macrophages and various T cell subsets, while some cell types show co-infiltration trends. **(g)** Boxplots comparing the fractions of key immune cells between control and treatment groups. Significant differences are observed in Tregs, M2 macrophages, and CD8 + T cells.

Figure 6 presents pathway enrichment analysis and immune cell profiling in relation to CXCL14 expression and experimental conditions. Panels (a) and (b) show GSEA plots, identifying pathways significantly enriched in samples with high and low CXCL14 expression, respectively. Pathways such as immune response and extracellular matrix organization are enriched in the high-expression group, whereas metabolic and proliferative pathways dominate the low-expression group. Panel (c) summarizes differentially enriched pathways between these groups, categorizing them as upregulated (green) or downregulated (orange). Panel (d) illustrates the relative proportions of immune cell types in control and treated groups, showing notable shifts in immune composition. In panel (e), a heatmap displays correlations between key genes (MMP7, MDK, CXCL14) and immune cell populations, with significant associations marked by asterisks. CXCL14 shows a strong positive correlation with macrophages and dendritic cells, whereas MMP7 is negatively associated with T cells. Panel (f) presents a correlation matrix of immune cell proportions, highlighting interactions between different immune cell types. Finally, panel (g) shows boxplots comparing immune cell fractions between control and treated groups, identifying significant differences in specific immune populations (Full details provided in Supplementary Table S6).

## 4 Discussion

In current study, SHAP analysis was applied in combination with machine learning to identify key genes associated with IPF, with a focus on immune-related genes. Differential gene expression analysis, after batch effects were corrected and the data were normalized, revealed a set of significantly dysregulated genes, with CXCL14 being identified as one of the most prominent. A logistic regression model based on these genes demonstrated high predictive accuracy, as indicated by a strong AUC in the ROC analysis. SHAP analysis further highlighted CXCL14 as the most influential feature in the model, with higher expression levels being strongly associated with positive SHAP values, confirming its critical role in IPF prediction. SHAP analysis was critical for translating machine learning results into biological insights, as it not only ranked feature importance (e.g., CXCL14 as the most influential gene) but also visualized interactions between genes (e.g., CXCL14 and TGF-β1), enabling us to deduce their combined roles in fibrosis and immune dysregulation. Functional enrichment analysis identified key biological processes and pathways related to immune responses and extracellular matrix remodeling, suggesting that the immune microenvironment plays a crucial role in IPF pathogenesis. Immune cell infiltration analysis also showed significant associations between CXCL14 expression and immune cell populations. These results confirm the importance of CXCL14 as a predictive biomarker and possibly a therapeutic target in IPF. The study illustrates how SHAP-based feature selection can enhance model interpretability, providing valuable insights into the molecular mechanisms underlying IPF.

In recent years, the regulatory mechanisms of the immune microenvironment in IPF have become a hotspot of research. Multiple studies have shown that chemokines such as CXCL9, CXCL10, and CXCL11 promote pulmonary fibrosis by recruiting fibrosis-related macrophages (31), but the role of CXCL14 in IPF has long been overlooked. Early studies reported upregulated expression of CXCL14 in lung fibroblasts (32), but its function was limited to promoting collagen deposition and did not involve immune regulation. In contrast, our study found that high expression of CXCL14 is significantly associated with the infiltration of CD4 + T cells and regulatory Treg (SHAP value = 0.43, *p* < 0.001), which resonates with the “chemokine-immune cell axis” theory proposed (33), but for the first time establishes a direct link between CXCL14 and adaptive immunity in IPF. It is noteworthy that our machine learning model revealed that the contribution of CXCL14 to IPF prediction (mean SHAP value = 0.38) far exceeds that of traditional biomarker MMP7 (mean SHAP value = 0.12), challenging the previous view that matrix metalloproteinases are the core driving factors of IPF (34). Furthermore, by comparing the GSE132607 and GSE213001 cohort data, we found that the expression of CXCL14 in progressive IPF patients is 2.3 times higher than that in stable patients (*p* = 0.008), which overlaps partially with the “disease progression-related gene cluster” characteristics reported (35), but our study further discovers a synergistic regulatory relationship between CXCL14 and TGF-β1 through SHAP interaction analysis (interaction SHAP value = 0.21), suggesting it may amplify pro-fibrotic signaling pathways. These findings provide a new perspective for re-understanding the immune-matrix cross-talk in IPF.

The core biological significance of this study is the revelation of the dual function of CXCL14: as an immunomodulator regulating the balance of T cell subgroups and as an activator of the PI3K/Akt/mTOR pathway (shown by KEGG enrichment analysis, FDR = 0.03) that promotes the transformation of fibroblasts into myofibroblasts. This dual mechanism may explain why local immune suppression and excessive fibrosis coexist in IPF-the high expression of CXCL14 may simultaneously induce Treg infiltration (inhibiting antifibrotic immune responses) and enhance fibroblast activation (promoting extracellular matrix deposition), a hypothesis highly consistent with the recently discovered “IPF-specific CXCL14 fibroblast subpopulation.” Clinically, CXCL14 shows significant diagnostic value: serum CXCL14 levels were strongly correlated with the rate of decline in forced vital capacity, with higher diagnostic sensitivity and specificity than the currently recommended KL-6 indicator (sensitivity 72%, specificity 65%) (36). More importantly, CXCL14 inhibitors (such as AMD3465) (37) reduced collagen deposition by 42% in an IPF mouse model (*p* = 0.01), providing preclinical evidence for the development of antibody drugs targeting CXCL14 (such as similar pirfenidone-like molecular design). Currently, monoclonal antibodies targeting the CXCL14/ACKR3 axis are in Phase I tumor clinical trials (NCT04857112), and our study provides a theoretical basis for expanding their indications to IPF.

Notably, this study primarily relies on bioinformatics analyses of publicly available datasets and lacks wet-lab validation of key findings, such as the functional roles of CXCL14 in immune cell infiltration or the mechanistic pathways identified by GSEA/KEGG enrichment. While the computational framework employed here is methodologically robust and provides a data-driven hypothesis for CXCL14’s dual role in fibrosis and immunity, experimental validation—such as *in vitro* cell culture assays or animal models—is essential to confirm causal relationships. For example, CRISPR-Cas9-mediated CXCL14 knockout in lung fibroblasts or adoptive transfer of Treg cells in IPF mouse models could mechanistically validate the predicted associations between CXCL14 expression and immune cell polarization.

Although this study has provided valuable findings, there are also some limitations. First, our data mainly come from public gene expression databases, which may be affected by batch effects and data integration issues. Second, although our predictive model shows good accuracy in ROC analysis, the generalizability of the model still needs to be validated through multi-center clinical samples with diverse ethnic and demographic backgrounds. Third, while SHAP analysis has enhanced the interpretability of the model, the complex interactions of some genes (e.g., CXCL14-TGF-β1 crosstalk) identified in silico require further verification through experimental research, such as co-immunoprecipitation or live-cell imaging. Looking forward, we intend to address these gaps in future studies by integrating wet-lab experiments, such as spatial transcriptomics to map CXCL14 expression in IPF lung tissue and functional assays to validate its receptor-mediated signaling pathways, once experimental resources become available. The follow-up study can delve into the following three dimensions: 1. Molecular Mechanism Analysis: Utilize spatial transcriptomics techniques (such as 10x Visium) to locate the expression pattern of CXCL14 in specific areas of IPF lung tissue (such as fibroblastic foci), combined with CRISPR interference technology to verify its spatial co-localization relationship with TGF-β1 (38, 39). 2. Precision Medicine Application: Establish a machine learning stratification model based on the expression level of CXCL14, integrate clinical parameters (such as glycomics and proteomics index) and radiomics features, and develop an IPF personalized prognostic prediction system (40). 3. Therapeutic Target Development: Screen small molecule compounds that specifically block the binding of CXCL14 to its receptor ACKR3 (similar to the design strategy of CCR5 inhibitor Maraviroc), and evaluate their antifibrotic effects in humanized IPF organoid models. At the same time, multi-center studies are needed to validate the expression heterogeneity of CXCL14 in different ethnicities, which is crucial for clinical applications worldwide.

## 5 Conclusion

This study identifies key differently DEGs and their functional roles through integrative bioinformatics analyses. Batch effect correction ensured data consistency, revealing DEGs enriched in immune modulation and extracellular matrix remodeling. Machine learning models highlighted MMP7, CXCL14, and MDK as critical biomarkers with strong predictive power. SHAP analysis confirmed their impact, while immune profiling uncovered key regulatory interactions. These findings provide insights into disease mechanisms and potential therapeutic targets. Future research should validate these biomarkers and explore their translational applications in precision medicine.

## Data Availability

The original contributions presented in the study are included in the article/Supplementary material, further inquiries can be directed to the corresponding author.

## Footnotes

^1^ https://www.ncbi.nlm.nih.gov/geo/

^2^ https://www.cancerrxgene.org/

## Glossary

IPF: Idiopathic Pulmonary Fibrosis
GEO: Gene Expression Omnibus
PCA: Principal Component Analysis
DEGs: Differentially Expressed Genes
LASSO: Least Absolute Shrinkage and Selection Operator
SHAP: Shapley Additive Explanations
TERT: Telomerase Mutations
MUC5B: Mucin 5B
Treg: Regulatory T Cells
DEA: Differentially Expressed Analysis
RF: Random Forest
CIBERSORT: Cell Type Identification by Estimating Relative Subsets of RNA Transcripts
GSEA: Gene Set Enrichment Analysis
GSVA: Gene Set Variation Analysis
KEGG: Kyoto Encyclopedia of Genes and Genomes
FDR: False Discovery Rate
GO: Gene Ontology
SVM: Support Vector Machine
ROC: Receiver Operating Characteristic
AUC: Area Under the Curve
BP: Biological Processes
MF: Molecular Functions
CC: Cellular Components
